# Vitamin D Deficiency at Hospital Admission With Community-Acquired Pneumonia is Associated With Increased Risk of Mortality: A Prospective Cohort Study

**DOI:** 10.1093/ofid/ofaf706

**Published:** 2025-11-19

**Authors:** Maria Hein Hegelund, Sehrash Alam, Arnold Matovu Dungu, Camilla Koch Ryrsø, Daniel Faurholt-Jepsen, Rikke Krogh-Madsen, Tomas Oestergaard Jensen, Christian Mølgaard, Birgitte Lindegaard

**Affiliations:** Department of Pulmonary and Infectious Diseases, Copenhagen University Hospital—North Zealand, Hillerød, Denmark; Department of Pulmonary and Infectious Diseases, Copenhagen University Hospital—North Zealand, Hillerød, Denmark; Department of Pulmonary and Infectious Diseases, Copenhagen University Hospital—North Zealand, Hillerød, Denmark; Department of Pulmonary and Infectious Diseases, Copenhagen University Hospital—North Zealand, Hillerød, Denmark; Centre for Physical Activity Research, Copenhagen University Hospital—Rigshospitalet, Copenhagen, Denmark; Department of Infectious Diseases, Copenhagen University Hospital—Rigshospitalet, Copenhagen, Denmark; Department of Clinical Medicine, University of Copenhagen, Copenhagen, Denmark; Centre for Physical Activity Research, Copenhagen University Hospital—Rigshospitalet, Copenhagen, Denmark; Department of Clinical Medicine, University of Copenhagen, Copenhagen, Denmark; Department of Infectious Diseases, Copenhagen University Hospital, Hvidovre, Copenhagen, Denmark; Department of Pulmonary and Infectious Diseases, Copenhagen University Hospital—North Zealand, Hillerød, Denmark; Centre of Excellence for Health, Immunity, and Infections, Rigshospitalet, University of Copenhagen, Copenhagen, Denmark; Department of Nutrition, Exercise and Sports, University of Copenhagen, Frederiksberg, Denmark; Department of Pulmonary and Infectious Diseases, Copenhagen University Hospital—North Zealand, Hillerød, Denmark; Centre for Physical Activity Research, Copenhagen University Hospital—Rigshospitalet, Copenhagen, Denmark; Department of Clinical Medicine, University of Copenhagen, Copenhagen, Denmark

**Keywords:** community-acquired pneumonia, mortality, vitamin D deficiency

## Abstract

**Background:**

A deficient vitamin D status is linked to increased risk of community-acquired pneumonia (CAP) and both short- and long-term mortality. Given small sizes of previous studies and lack of adjustment for key confounders, we aimed to investigate the association between vitamin D status (sufficient, insufficiency, or deficiency) and mortality risk in adults hospitalized with CAP.

**Methods:**

This study, nested within the Surviving Pneumonia Study at Copenhagen University Hospital—North Zealand, Denmark, included adults hospitalized with CAP between 2019 and 2022. Vitamin D status was assessed using serum 25(OH)D concentrations, categorizing participants as sufficient (≥50 nmol/L), insufficient (25–<50 nmol/L), or deficient (<25 nmol/L). Logistic regression was used to assess mortality risk. Covariates included age, sex, Charlson comorbidity index, CURB-65, smoking history, and BMI.

**Results:**

Among 514 participants, 29 (5.6%) and 130 (25.3%) had deficient and insufficient vitamin D status, respectively. Participants with deficient vitamin D status were younger, and more than 50% were current smokers. Vitamin D deficiency was associated with higher 90-day (OR: 3.50, 95% CI 1.01; 12.21) and 180-day (OR: 3.27, 95% CI 1.04; 10.25) mortality risk compared with participants with sufficient vitamin D status, while no difference was observed between the sufficient and insufficient group. No differences were observed for in-hospital or 30-day mortality.

**Conclusions:**

Participants with deficient vitamin D status were younger and faced higher mortality risk despite milder disease at admission. Given that vitamin D deficiency may relate to poorer health habits and low levels of other micronutrients, trials on tailored micronutrient supplementation during acute conditions like CAP could be considered.

## Teaser

A deficient vitamin D status was linked to increased 90- and 180-day mortality in adults hospitalized with CAP, despite milder disease upon admission. No association was found for insufficient vitamin D status. Results support exploring micronutrient supplementation during acute illness.

Vitamin D deficiency is common in many populations [1], especially in the winter season with limited exposure to sunlight. Vitamin D is a fat-soluble vitamin produced by the skin when exposed to sunlight, but also present in food like oily fish, eggs, and fortified products [2]. Vitamin D is crucial for the human body, including calcium absorption, bone metabolism, cardiovascular function, muscle function, and hormone regulation [3]. Vitamin D also plays a role in the regulation of the immune system, and evidence suggests that vitamin D deficiency increases the risk of respiratory tract infections, including community-acquired pneumonia (CAP) [4–6]. CAP is a global health challenge leading to high morbidity and mortality [7], especially among the elderly and individuals with chronic diseases [8, 9]. In addition to the increased risk of developing CAP [4–6], vitamin D deficiency has also been associated with a higher likelihood of hospitalization with CAP [10]. In observational studies among hospitalized individuals, low vitamin D levels have also been associated with CAP severity [10–12 ], ICU admission [12, 13], and length of hospital stay [11], as well as short-term (28-day or 30-day) and long-term (5-year) mortality among hospital survivors [12–17].

Previous studies have mainly included relatively small samples without adjustment for important confounders in the statistical models, such as body mass index (BMI), smoking history [12, 13, 15], and comorbidities [13, 15]. Further, these studies did not investigate mortality beyond 30 days after discharge [12, 13, 15], except for one study investigating 5-year mortality [17]. We hypothesized that vitamin D deficiency would be prevalent among adults hospitalized with CAP and that vitamin D deficiency at admission was associated with a higher risk of 90-day mortality. The overall aim of this study was to investigate the potential association between low vitamin D concentrations (insufficiency and deficiency) and the risk of 90-day mortality (primary outcome) as well as in-hospital, 30-day, and 180-day mortality (secondary outcomes).

## METHODS

### Study Design, Setting, and Population

This study used data from the Surviving Pneumonia Study cohort (ClinicalTrials.gov: NCT03795662), which is an ongoing prospective cohort study of adults (≥18 years) hospitalized with CAP at Copenhagen University Hospital—North Zealand, Denmark. Enrollment was conducted within 24 hours of admission from the emergency department and medical wards, and the inclusion period for this study was between January 2019 and April 2022. The follow-up period was 180 days from the day of admission. The inclusion criteria for this study were age ≥18 years and a new pulmonary infiltrate on chest X-ray/Computed Tomography scan and minimum one of the following symptoms: fever (≥38.0°C), cough, pleuritic chest pain, dyspnea, or focal chest signs on auscultation. The exclusion criteria for the present study were no biobank sample for measurement of serum 25-hydroxyvitamin D (25(OH)D) concentration and participation in an intervention study.

### Data Collection

Information related to socio-demography, lifestyle, anthropometry, and comorbidities, as well as clinical and paraclinical data, was collected within 48 hours of admission through interviews, measurements, questionnaires, and medical records.

### Lifestyle and Anthropometry

Patients were categorized according to their smoking history as never, previous, and current smokers. Alcohol usage was categorized as an intake according to or above recommendations by the Danish authorities at that time, ie, a maximum intake of 7 units/week for females and 14 units/week for males [18]. Weight was measured within 48 hours after admission to the nearest 0.1 kg on an electronic scale (Seca, Hamburg, Germany), height was self-reported, and BMI was calculated as weight (kg)/height (m^2^).

### Laboratory and Clinical Data

Blood samples were collected at admission and analyzed by the Department of Clinical Biochemistry, Copenhagen University Hospital—North Zealand for levels of C-reactive protein (mg/L), albumin (g/L), hemoglobin (nmol/L), B12 (pmol/L), folate (nmol/L), and iron (µmol/L). For analysis of 25(OH)D levels, blood samples were drawn in EDTA tubes and stored on ice until centrifuged at 3000 *g* for 15 minutes at 4°C. Serum was separated and stored at −80°C until analyzed by the Department of Clinical Biochemistry, Copenhagen University Hospital—North Zealand with the commercially available ADVIA Centaur^®^ Vitamin D Total (Siemens Healthcare Diagnostics Inc., Erlangen, Germany). The detection limit was below 11 nmol/L. Patients were categorized according to their serum 25 (OH)D concentrations as sufficient (≥50 nmol/L), insufficient (25–<50 nmol/L), or deficient vitamin D status (<25 nmol/L) [19]. The comorbidity burden was assessed with the Charlson comorbidity index, with a higher score indicating higher mortality risk [20]. Pneumonia severity was assessed using the CURB-65 score, where the scoring is based on the following parameters: level of confusion, carbamide ≥ 7 mmol/L, respiratory rate over 30 breaths/min, blood pressure (diastolic ≤ 60 mm Hg or systolic < 90 mm Hg), and age ≥ 65 years. Every parameter gives 1 point, and the total score categorizes pneumonia severity as mild (0–1), moderate (2), and severe (3–5) [21].

### Outcomes

The primary outcome was 90-day mortality. The secondary outcomes were in-hospital, 30-day, and 180-day mortality. Information on mortality was collected through medical records and the follow-up period was 180 days from the day of admission.

### Ethical Considerations

The Surviving Pneumonia Study was approved by the Scientific Ethics Committee at the Capital Region of Denmark (H-18024256), registered on ClinicalTrials.gov (NCT03795662), and conducted in accordance with the Declaration of Helsinki [22]. All the subjects were made aware of the protocol and the purpose of the study. Oral and written consents were taken from the study participants before enrollment in the study.

### Statistical Methods

Statistical analyses were carried out using STATA/IC version 17.0 (StataCorp LP, College Station, TX, USA). The distribution of all variables was evaluated, and the data were reported as means with standard deviations (SD) for normally distributed variables, median with interquartile range (IQR) for non-normally distributed variables, and count (%) for categorical variables.

The association between vitamin D status and risk of mortality (in-hospital, 30-day, 90-day, and 180-day) was evaluated using logistic regression. Sufficient vitamin D concentration was used as the reference group in the analyses. Predefined covariates included age, sex, Charlson comorbidity index, CURB-65, smoking history, and BMI. The main analysis was an adjusted model with imputed data, as several covariates had missing values. Missing variables were imputed using chained equations, creating 10 imputed data sets with 100 iterations per dataset [23 , 24 ]. The imputation included the following variables: age, sex, mortality, Charlson comorbidity index, smoking status, CURB-65, BMI, and vitamin D status. In addition, unadjusted and adjusted complete case models were conducted for each outcome. Estimates shown are odds ratios (OR) with corresponding 95% confidence intervals (CI) and *P*-values. The results were considered significant when the *P*-value was <.05.

## RESULTS

**Figure 1.**
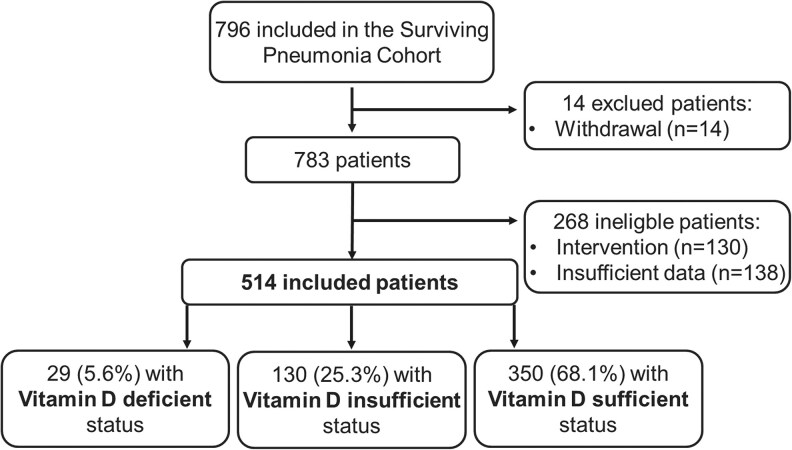
Flowchart showing participant selection and categorization by vitamin D status in the *Surviving Pneumonia Study*.

Of the 796 adults admitted with CAP in the study period, 514 (65%) were eligible for the present study. Of the ineligible individuals, 14 (2%) were excluded due to withdrawal of consent, 130 (16%) due to participation in an intervention study, and 138 (17%) had no biobank sample to analyze for serum 25(OH)D level (Figure 1). There were a lower proportion of females and a slightly lower Charlson comorbidity index among participants compared to nonparticipants, but no difference regarding CAP severity and mortality (data not shown).

### Baseline Characteristics Across Vitamin D Groups

The baseline and outcome characteristics are shown in Table 1. Of the study population, 29 participants (5.6%) had a deficient vitamin D status, whereas 130 (25.2%) and 350 (68%) had insufficient and sufficient vitamin D status, respectively. The median (IQR) age was 74 (63–81) years, 228 (44%) were females, 63 (14%) had severe CAP, and the median (IQR) length of stay was 5.4 (3.4–9.0) days.

**Table 1.** Baseline Characteristics and Outcome Measures According to Vitamin D Status Among Patients Hospitalized With Community-Acquired Pneumonia

	Total Sample (*n* = 514)	Deficient <25 (*n* = 29)	Insufficient 25–50 (*n* = 130)	Sufficient ≥ 50 (*n* = 350)
Age, y	74 (63–81)	64 (57–72)	71.5 (62–80)	75 (66–81)
Female sex	228 (44.4)	10 (34.5)	51 (39.2)	167 (47.0)
BMI, kg/m^2^	25.9 (22.4–29.4)	27.5 (22.5–29.8)	25.9 (23.0–29.1)	25.8 (22.3–29.4)
<18.5	20 (5.1)	2 (8.3)	4 (3.9)	14 (5.3)
18.5–24.9	148 (37.8)	6 (25.0)	40 (38.5)	102 (38.6)
25–29.9	140 (35.7)	10 (41.7)	37 (35.6)	93 (35.2)
≥30	84 (21.4)	6 (25.0)	23 (22.1)	55 (20.8)
Alcohol intake				
Recommended	396 (77.0)	23 (79.3)	104 (80.0)	269 (75.8)
>Recommended	74 (14.4)	5 (17.2)	15 (11.5)	54 (15.2)
Unknown	44 (8.6)	1 (3.5)	11 (8.5)	32 (9.0)
Smoking status				
Never	124 (26.6)	3 (12.0)	31 (26.1)	90 (27.9)
Previous	268 (57.4)	8 (32.0)	70 (58.8)	190 (58.8)
Current	75 (16.1)	14 (56.0)	18 (15.1)	43 (13.3)
Comorbidities				
Charlson comorbidity index	4 (3–6)	3 (2–5)	4 (2–6)	4 (3–6)
Chronic obstructive pulmonary disease	175 (34.1)	12 (41.4)	37 (28.5)	126 (35.5)
Cardiovascular diseases	162 (31.5)	6 (20.7)	43 (33.1)	113 (31.8)
Diabetes	90 (17.5)	6 (20.7)	21 (16.2)	63 (17.8)
Cancer	93 (18.1)	3 (10.3)	22 (16.9)	68 (19.2)
Laboratory characteristics				
C-reactive protein, mg/L	97.4 (37–163.6)	82.1 (48.4–159.2)	107.7 (46.2–154.2)	97.1 (36.3–168.3)
Albumin, g/L	28.6 ± 5.1	30.0 ± 5.8	28.7 ± 5.0	28. ± 5.1
Hemoglobin, mmol/L	7.9 ± 1.3	8.3 ± 1.5	7.9 ± 1.5	7.8 ± 1.3
B12, pmol/L	577.1 (392.3–966.8)	418.8 (298.7–700.7)	503.4 (371.0–782.3)	639.7 (409.5–1019.0)
Folate, nmol/L	24.9 (16.7–36.9)	16.0 (6.7–20.6)	20.8 (12.8–27.4)	29.9 (19.8–40.7)
Iron, µmol/L	4.9 (3.7–8.3)	4.5 (3.9–8.2)	4.5 (3.2–7.4)	5.0 (3.8–8.6)
Severity characteristics				
CURB-65 index^a^				
Mild	87 (18.8)	8 (33.3)	28 (24.1)	51 (15.7)
Moderate	314 (67.7)	16 (66.7)	76 (65.5)	222 (68.5)
Severe	63 (13.6)	0 (0.0)	12 (10.3)	51 (15.7)
Length of stay	5.4 (3.4–9.0)	6.4 (3.5–10.5)	5.4 (3.3–9.0)	5.3 (3.4–8.9)
ICU admission	36 (7.0)	0 (0.0)	13 (10.0)	23 (6.5)
Mortality				
In-hospital mortality	40 (7.8)	2 (6.9)	11 (8.5)	27 (7.6)
30-d	55 (10.7)	3 (10.3)	15 (11.5)	37 (10.4)
90-d	80 (15.7)	5 (17.2)	24 (18.5)	51 (14.4)
180-d	98 (19.1)	6 (20.1)	28 (21.5)	64 (18.0)

Data are presented as median (IQR), mean ± SD and *n* (%).

^a^Assessed using the CURB-65 scoring system including confusion (yes/no), urea (>7 mmol/L), respiratory rate (≥30/min), blood pressure (systolic <90 mm Hg or diastolic ≤60 mm Hg), and age (≥65 y).

Vitamin D status was categorized as sufficient (≥50 nmol/L), insufficient (25–<50 nmol/L), or deficient vitamin D (<25 nmol/L) according to their serum 25 (OH)D concentration.

Available data: Age (*n* = 514), sex (*n* = 514), BMI (*n* = 392), alcohol intake (*n* = 514), smoking status (*n* = 467), Charlson comorbidity index (*n* = 514), C-reactive protein (*n* = 510), albumin (*n* = 474), hemoglobin (*n* = 441), B12 (*n* = 431), Folate (*n* = 427), Iron (*n* = 368), and CURB-65 (*n* = 464).

Those with deficient vitamin D status were younger, with a median (IQR) age of 64 (57–62) years, compared to 71.5 (62–80) and 75 (66–81) years among those with insufficient and sufficient vitamin D status. Compared to the two other groups, those with deficient vitamin D status had lower levels of vitamin B12 (deficient: 419 pmol/L (299–701); insufficient: 503 pmol/L (371–782); sufficient: 640 pmol/L (410–1019) and folate (deficient: 16.0 nmol/L (6.7–20.6); insufficient: 20.8 nmol/L (12.8–27.4); sufficient: 29.9 nmol/L (19.8–40.7)). The median (IQR) Charlson comorbidity indices were 3 (2–5), 4 (2–6), and 4 (3–6) among participants with deficient, insufficient, and sufficient vitamin D status, respectively. Among participants with deficient vitamin D status, 56% were current smokers compared to 15% and 13% among participants with insufficient and sufficient vitamin D status. In addition, participants with deficient vitamin D status had milder CAP compared to the two other groups, with 67%, 76%, and 84% categorized as moderate to severe CAP, according to CURB-65, among participants with deficient, insufficient, and sufficient vitamin D status, respectively. Further no participants with deficient vitamin D status were admitted to the intensive care unit compared to 10% and 6.5% among participants with insufficient and sufficient vitamin D status (Table 1).

The overall in-hospital, 30-day, 90-day, and 180-day mortality rates were 7.8%, 10.7%, 15.7%, and 19.1%.

Among the 514 included participants, 179 (35%) had incomplete data for minimum one covariate variable. There were no differences in age, sex, Charlson comorbidity index, or in-hospital mortality between participants with complete and incomplete data. However, participants with complete data had lower 30-day (8.4% vs 15.1%), 90-day (12.4% vs 20.7%), and 180-day (16.4% vs 24.0%) mortality rates compared to participants with incomplete data (data not shown).

### Risk of 90-Day Mortality (Primary Outcome)

Those with deficient vitamin D status had a higher risk of 90-day mortality compared to those with sufficient vitamin D status (OR 3.50, 95% CI 1.01; 12.21), while there was no difference in 90-day mortality risk between those with insufficient and sufficient vitamin D status (OR 1.73, 95% CI 0.94; 3.20) (Table 2).

**Table 2.** Risk of In-hospital, 30-Day, 90-Day, and 180-Day Mortality by Vitamin D Status Among Patients Hospitalized With Community-acquired Pneumonia

Mortality	Model 1 (unadjusted)		Model 2 (adjusted Complete Case)		Model 3 (adjusted—imputed Data)	
	OR (95% CI)	*P*-value	OR (95% CI)	*P*-value	OR (95% CI)	*P*-value
In-hospital						
Vitamin D sufficient	Ref.		Ref.	…	Ref.	…
Vitamin D insufficient	1.12 0.54; 2.33)	.76	0.93 (0.31; 2.82)	.90	1.32 (0.59; 2.96)	.51
Vitamin D deficient	0.90 (0.20; 3.99)	.89	-	…	2.07 (0.39; 11.11)	.39
30-d						
Vitamin D sufficient	Ref.		Ref.	…	Ref.	…
Vitamin D insufficient	1.12 (0.59; 3.44)	.73	1.14 (0.42; 3.06)	.80	1.37 (0.66; 2.84)	.46
Vitamin D deficient	0.99 (0.29; 3.44)	.99	1.19 (0.12; 12.27)	.88	2.46 (0.54; 11.13)	.24
90-d						
Vitamin D sufficient	Ref.		Ref.	…	Ref.	…
Vitamin D insufficient	1.35 (0.79; 2.30)	.27	1.58 (0.77; 17.53)	.26	1.73 (0.94; 3.20)	.08
Vitamin D deficient	1.24 (0.45; 3.40)	.67	3.67 (0.77; 17.53)	.10	3.50 (1.01; 12.21)	.049
180-d						
Vitamin D sufficient	Ref.		Ref.	…	Ref.	…
Vitamin D insufficient	1.25 (0.76; 2.05)	.38	1.37 (0.65; 2.89)	.41	1.52 (0.86; 2.71)	.15
Vitamin D deficient	1.19 (0.46; 0.03)	.72	3.92 (0.96; 16.38)	.06	3.27 (1.04; 10.25)	.04

Cox regression was used to analyze risk of re-hospitalization with mortality as competing event. Logistic regression was used to analyze risk of mortality. Estimates shown are hazard ratio (HR) and odds ratios (OR) with corresponding 95% confidence intervals (CI) and *P*-value. Model 1: unadjusted. Model 2: complete case analysis adjusted for age, sex, Charlson Comorbidity Index, CURB-65, smoking and BMI. Model 3: adjusted for age, sex, Charlson Comorbidity Index, CURB-65, smoking and BMI with imputed values for covariate with missing data. Values were imputed in CURB-65 (*n* = 50), smoking (*n* = 47), and BMI (*n* = 122).

### Risk of in-hospital, 30-Day, and 180-Day Mortality

There was no difference in risk of in-hospital (OR 1.32, 95% CI 0.59; 2.96), 30-day (OR 1.37, 95% CI 0.66; 2.84), or 180-day (OR 1.52, 95% CI 0.86; 2.71) mortality between those with sufficient vitamin D status and those with insufficient status. Those with deficient vitamin D status had a higher risk of 180-day mortality compared to those with sufficient vitamin D status (OR 3.27, 95% CI 1.04; 10.25), whereas there was no difference in risk of in-hospital (OR 2.07, 95% CI 0.39; 11.11) or 30-day (OR 2.46, 95% CI 0.54; 11.13) mortality between participants with deficient and sufficient vitamin D status.

## DISCUSSION

Our results showed that among adults hospitalized with CAP, a deficient vitamin D status was associated with a higher risk of 90-day and 180-day mortality after adjusting for age, sex, Charlson comorbidity index, smoking history, BMI, and initial CAP severity indicated by CURB-65 score.

In our study population, the prevalence of vitamin D deficiency was surprisingly low as only 6% had a deficient vitamin D status. Furthermore, participants with a deficient vitamin D status were younger than those with insufficient or sufficient vitamin D status. This finding may, to some extent, be explained by adherence to the vitamin D supplementation recommendations by the Danish Health Authority. For many years, the Danish Health Authority has recommended vitamin D supplementation for certain population groups, including the elderly above 64 years. However, in December 2020, the recommendations were updated, and the current guidelines advice all individuals (children and adults of all ages) to take vitamin D supplements during the winter season (October–April) to ensure adequate serum 25(OH)D concentrations [25].

The prevalence of a deficient vitamin D status in our population was markedly lower than the prevalence of 15–80% reported in other studies [12–15]. In one study, patients taking any type of vitamin D supplements were excluded [14], whereas a few studies excluded individuals taking specific types of vitamin D supplements recommended for certain medical conditions, such as kidney disease, where the body's ability to convert vitamin D into its active form is impaired [12, 13]. These specialized supplements are not commonly used by the general population. A study of vitamin D status, intake, and recommendations across European countries reported significant variations in serum (25(OH)D) concentrations likely due to differences in sunlight exposure, cultural habits, food fortification policies, and adherence to recommendations from health authorities [26].

Individuals with deficient vitamin D status may represent a population with worse health habits, as indicated in our study by lower levels of other vitamins (B12 and folate) and a higher proportion of current smokers. This finding is supported by another study in a general European population (aged 37–73 years at inclusion), which reported a higher proportion of physically active individuals and nonsmokers among those with higher vitamin D status [27].

In our study, a deficient vitamin D status was associated with increased risk of 90-day and 180-day mortality. We could not detect an association between a deficient vitamin D status and in-hospital or 30-day mortality. The estimates from the statistical model indicate a possibility of increased risk for in-hospital and 30-day mortality, though the results are far from statistically significant. In previous studies, a deficient vitamin D status has been associated with an increased risk of short-term mortality such as 28-day or 30-day mortality, among individuals hospitalized with CAP [12, 14, 15]. The lacking association between short-term mortality and a deficient vitamin D status in our population may be due to the low number of events (ie, deaths) among the participants with deficient vitamin D status, as only two and three patients died during admission and within 30 days from admission, respectively. Studies of nonhospitalized individuals have also reported that a deficient vitamin D status was associated with an increased risk of long-term mortality, including both all-cause mortality and death from respiratory diseases such as CAP, with follow-up periods of up to 14 years [27, 28].

The main strength of this study is the availability of data on important potential confounders, including smoking history, BMI, and comorbidities. None of the other identified studies investigating the association between a deficient vitamin D status and mortality among adults hospitalized with CAP included smoking or BMI as potential confounders [12, 13, 15] and only one study included comorbidities as a potential confounder [12]. Not including potential confounders could result in misleading results. In these cases, the association between a deficient vitamin D status and short-term mortality could be overestimated or misleading if it was attributed to other factors not included in the model. Another strength is the relatively large sample size. One limitation of our study was the low number of events related to in-hospital and 30-day mortality. The inability to detect a potential association between a deficient vitamin D status and these outcomes could be due to a lack of statistical power. The risk of selection bias is another potential limitation, as those with the most severe disease may decline to participate. In addition, it is likely that among included patients, the most severely ill may dropout more frequently compared to those with milder disease. To minimize the lack of follow-up data due to attrition bias, participants were given the option to consent only to use their medical record for future follow-up if they expressed a desire to withdraw their consent due to their condition. Missing data on several variables considered important potential confounders is another potential limitation. We used imputation, which is a valid statistical model, to enable a more powerful multivariate model adjusting for potential confounders.

Vitamin D status and supplementation have been debated for many years without reaching a consensus on the role of supplementation, the optimal dose, or the optimal 25(OH)D concentration as indicator of a sufficient vitamin D status. Poor trial designs and dose heterogeneity may explain the lack of evidence for benefit [29]. Further, most studies on vitamin D supplementation have not only focused on individuals who are depleted. It has been suggested that 25(OH)D concentration act as an acute-phase reactant, with low concentration during acute illness reflecting an immune response [30]. Therefore, vitamin D status should be interpreted with caution during acute illness. A systematic review on COVID-19 found lower concentration of 25(OH)D in individuals with severe disease compared to those with milder forms [31]. However, our observation of milder disease and lower C-reactive protein levels in participants with deficient vitamin D status suggests true deficiency rather than just an immune response.

There remains some debate regarding the optimal 25(OH)D concentration that defines sufficiency. While a threshold of 50 nmol/L is widely accepted, several large population-based studies of chronic conditions and all-cause mortality have suggested that mortality risk continues to decline at higher concentrations, up to ∼75 nmol/L or above [32, 33]. In our cohort, exploratory analyses did not indicate any further reduction in mortality above this concentration, which is consistent with the notion that the association between vitamin D status and mortality may plateau once concentrations exceed about 50 nmol/L. Studies conducted in acute infectious diseases, including COVID-19, have similarly not demonstrated additional benefit at high 25(OH)D concentrations, although a deficient vitamin D status is consistently associated with poorer outcomes [34, 35]. Together, these findings suggest that maintaining serum 25(OH)D concentrations above 50 nmol/L is likely sufficient for reducing mortality risk, with limited evidence of further advantage at higher concentrations in either chronic or acute conditions.

A large multicenter double-blind placebo randomized controlled trial (aiming at 2400 participants) is currently recruiting participants. This study will investigate the effect of high-dose vitamin D supplementation on 28-day mortality among critically ill patients [36] and contribute to the question of causality. Our results and those from other studies [27, 28] indicate that maintaining serum 25(OH)D concentrations above 50 nmol/L may be beneficial for reducing mortality risk among both hospitalized and nonhospitalized individuals.

## CONCLUSION

Our results show that participants with a deficient vitamin D status were relatively younger and faced an increased mortality risk despite milder disease at admission. Since a deficient vitamin D status may be associated with poorer health habits, including low levels of other micronutrients, trials investigating the effects of supplementing with tailored micronutrient combinations during acute conditions like CAP could be considered to assess the effect on mortality.

## References

[1] Holick MF, Chen TC. Vitamin D deficiency: a worldwide problem with health consequences. Am J Clin Nutr 2008; 87:1080s–6s. 18400738 10.1093/ajcn/87.4.1080S

[2] National Institutes of Health . Vitamin D. Factsheet for Helath Professionals. [updated June 27, 2025; cited 2025 October 10]. Available at: https://ods.od.nih.gov/factsheets/VitaminD-HealthProfessional/.

[3] Khammissa RAG, Fourie J, Motswaledi MH, Ballyram R, Lemmer J, Feller L. The biological activities of vitamin D and its receptor in relation to calcium and bone homeostasis, cancer, immune and cardiovascular systems, skin biology, and oral health. Biomed Res Int 2018; 2018:9276380. 29951549 10.1155/2018/9276380 PMC5987305

[4] Zhou YF, Luo BA, Qin LL. The association between vitamin D deficiency and community-acquired pneumonia: a meta-analysis of observational studies. Medicine (Baltimore) 2019; 98:e17252. 31567995 10.1097/MD.0000000000017252 PMC6756683

[5] Aregbesola A, Voutilainen S, Nurmi T, Virtanen JK, Ronkainen K, Tuomainen TP. Serum 25-hydroxyvitamin D3 and the risk of pneumonia in an ageing general population. J Epidemiol Community Health 2013; 67:533–6. 23596250 10.1136/jech-2012-202027

[6] Lu D, Zhang J, Ma C, et al Link between community-acquired pneumonia and vitamin D levels in older patients. Z Gerontol Geriatr 2018; 51:435–9. 28477055 10.1007/s00391-017-1237-z

[7] Ferreira-Coimbra J, Sarda C, Rello J. Burden of community-acquired pneumonia and unmet clinical needs. Adv Ther 2020; 37:1302–18. 32072494 10.1007/s12325-020-01248-7 PMC7140754

[8] Huang D, He D, Gong L, et al Clinical characteristics and risk factors associated with mortality in patients with severe community-acquired pneumonia and type 2 diabetes mellitus. Crit Care 2021; 25:419. 34876193 10.1186/s13054-021-03841-w PMC8650350

[9] Corrales-Medina VF, Musher DM, Wells GA, Chirinos JA, Chen L, Fine MJ. Cardiac complications in patients with community-acquired pneumonia: incidence, timing, risk factors, and association with short-term mortality. Circulation 2012; 125:773–81. 22219349 10.1161/CIRCULATIONAHA.111.040766

[10] Jovanovich AJ, Ginde AA, Holmen J, et al Vitamin D level and risk of community-acquired pneumonia and sepsis. Nutrients 2014; 6:2196–205. 24918697 10.3390/nu6062196 PMC4073143

[11] Mudipalli D . Correlational study of vitamin-D deficiency levels and its severity of community-acquired pneumonia in patients admitted into a tertiary care hospital. J Appl Pharmaceut Res 2024; 12:51–6.

[12] Leow L, Simpson T, Cursons R, Karalus N, Hancox RJ. Vitamin D, innate immunity and outcomes in community acquired pneumonia. Respirology 2011; 16:611–6. 21244571 10.1111/j.1440-1843.2011.01924.x

[13] Remmelts HH, van de Garde EM, Meijvis SC, et al Addition of vitamin D status to prognostic scores improves the prediction of outcome in community-acquired pneumonia. Clin Infect Dis 2012; 55:1488–94. 22942205 10.1093/cid/cis751

[14] Talebi F, Rasooli Nejad M, Yaseri M, Hadadi A. Association of vitamin D status with the severity and mortality of community-acquired pneumonia in Iran during 2016–2017: a prospective cohort study. Rep Biochem Mol Biol 2019; 8:85–90. 31334293 PMC6590933

[15] Kim HJ, Jang JG, Hong KS, Park JK, Choi EY. Relationship between serum vitamin D concentrations and clinical outcome of community-acquired pneumonia. Int J Tuberc Lung Dis 2015; 19:729–34. 25946368 10.5588/ijtld.14.0696

[16] Moraes RB, Friedman G, Wawrzeniak IC, et al Vitamin D deficiency is independently associated with mortality among critically ill patients. Clinics (Sao Paulo) 2015; 70:326–32. 26039948 10.6061/clinics/2015(05)04 PMC4449478

[17] Holter JC, Ueland T, Norseth J, et al Vitamin D status and long-term mortality in community-acquired pneumonia: secondary data analysis from a prospective cohort. PLoS One 2016; 11:e0158536. 27367810 10.1371/journal.pone.0158536 PMC4930204

[18] Sundhedsstyrelsen . Forebyggelsespakke—Alkohol. Copenhagen: Sundhedsstyrelsen. 2018.

[19] Mosekilde L, Nielsen LR, Larsen ER, Moosgaard B, Heickendorff L. [Vitamin D deficiency. Definition and prevalence in Denmark]. Ugeskr Laeger 2005; 167:29–33. 15675161

[20] Quan H, Li B, Couris CM, et al Updating and validating the Charlson comorbidity index and score for risk adjustment in hospital discharge abstracts using data from 6 countries. Am J Epidemiol 2011; 173:676–82. 21330339 10.1093/aje/kwq433

[21] Lim WS, van der Eerden MM, Laing R, et al Defining community acquired pneumonia severity on presentation to hospital: an international derivation and validation study. Thorax 2003; 58:377–82. 12728155 10.1136/thorax.58.5.377 PMC1746657

[22] World Medical Association . World Medical Association Declaration of Helsinki: ethical principles for medical research involving human subjects. JAMA 2013; 310:2191–4. 24141714 10.1001/jama.2013.281053

[23] Marshall A, Altman DG, Royston P, Holder RL. Comparison of techniques for handling missing covariate data within prognostic modelling studies: a simulation study. BMC Med Res Methodol 2010; 10:7. 20085642 10.1186/1471-2288-10-7 PMC2824146

[24] Azur MJ, Stuart EA, Frangakis C, Leaf PJ. Multiple imputation by chained equations: what is it and how does it work? Int J Methods Psychiatr Res 2011; 20:40–9. 21499542 10.1002/mpr.329 PMC3074241

[25] Sundhedsstyrelsen . Anbefalinger om D-vitamin og calcium [Internet]. 2024. Available at: https://www.sst.dk/da/Borger/En-sund-hverdag/Kost/D-vitamin-og-calcium.

[26] Spiro A, Buttriss JL. Vitamin D: an overview of vitamin D status and intake in Europe. Nutr Bull 2014; 39:322–50. 25635171 10.1111/nbu.12108 PMC4288313

[27] Sutherland JP, Zhou A, Hyppönen E. Vitamin D deficiency increases mortality risk in the UK biobank: a nonlinear Mendelian randomization study. Ann Intern Med 2022; 175:1552–9. 36279545 10.7326/M21-3324

[28] Wang TY, Wang HW, Jiang MY. Prevalence of vitamin D deficiency and associated risk of all-cause and cause-specific mortality among middle-aged and older adults in the United States. Front Nutr 2023; 10:1163737. 37275650 10.3389/fnut.2023.1163737 PMC10232798

[29] Amrein K, Scherkl M, Hoffmann M, et al Vitamin D deficiency 2.0: an update on the current status worldwide. Eur J Clin Nutr 2020; 74:1498–513. 31959942 10.1038/s41430-020-0558-y PMC7091696

[30] Waldron JL, Ashby HL, Cornes MP, et al Vitamin D: a negative acute phase reactant. J Clin Pathol 2013; 66:620–2. 23454726 10.1136/jclinpath-2012-201301

[31] Pereira M, Dantas Damascena A, Galvão Azevedo LM, de Almeida Oliveira T, da Mota Santana J. Vitamin D deficiency aggravates COVID-19: systematic review and meta-analysis. Crit Rev Food Sci Nutr 2022; 62:1308–16. 33146028 10.1080/10408398.2020.1841090

[32] Khaw KT, Luben R, Wareham N. Serum 25-hydroxyvitamin D, mortality, and incident cardiovascular disease, respiratory disease, cancers, and fractures: a 13-y prospective population study. Am J Clin Nutr 2014; 100:1361–70. 25332334 10.3945/ajcn.114.086413 PMC4196486

[33] Song S, Lyu J, Song BM, Lim JY, Park HY. Serum 25-hydroxyvitamin D levels and risk of all-cause and cause-specific mortality: a 14-year prospective cohort study. Clin Nutr 2024; 43:2156–63. 39142109 10.1016/j.clnu.2024.07.049

[34] Nielsen NM, Junker TG, Boelt SG, et al Vitamin D status and severity of COVID-19. Sci Rep 2022; 12:19823. 36396686 10.1038/s41598-022-21513-9 PMC9672358

[35] Akbar MR, Wibowo A, Pranata R, Setiabudiawan B. Low serum 25-hydroxyvitamin D (Vitamin D) level is associated with susceptibility to COVID-19, severity, and mortality: a systematic review and meta-analysis. Front Nutr 2021; 8:660420. 33855042 10.3389/fnut.2021.660420 PMC8039288

[36] Amrein K, Parekh D, Westphal S, et al Effect of high-dose vitamin D3 on 28-day mortality in adult critically ill patients with severe vitamin D deficiency: a study protocol of a multicentre, placebo-controlled double-blind phase III RCT (the VITDALIZE study). BMJ Open 2019; 9:e031083. 10.1136/bmjopen-2019-031083 PMC6858186 31722941

